# Sumoylation of the Rad1 nuclease promotes DNA repair and regulates its DNA association

**DOI:** 10.1093/nar/gku300

**Published:** 2014-04-20

**Authors:** Prabha Sarangi, Zdenka Bartosova, Veronika Altmannova, Cory Holland, Melita Chavdarova, Sang Eun Lee, Lumir Krejci, Xiaolan Zhao

**Affiliations:** 1 Molecular Biology Program, Memorial Sloan-Kettering Cancer Center, New York, NY 10065, USA; 2 Programs in Biochemistry, Cell, and Molecular Biology, Weill Cornell Graduate School of Medical Sciences, New York, NY 10065, USA; 3 Department of Biology, Masaryk University, Brno 62500, Czech Republic; 4 Department of Molecular Medicine, Institute of Biotechnology, University of Texas Health Science Center at San Antonio, San Antonio, TX 78229, USA; 5 National Centre for Biomolecular Research, Masaryk University, Brno 62500, Czech Republic; 6 Division of Radiation Biology, Department of Radiation Oncology, University of Texas Health Science Center at San Antonio, San Antonio, TX 78229, USA; 7 International Clinical Research Center, St. Anne's University Hospital in Brno, Brno 60200, Czech Republic

## Abstract

The *Saccharomyces cerevisiae* Rad1-Rad10 complex is a conserved, structure-specific endonuclease important for repairing multiple types of DNA lesions. Upon recruitment to lesion sites, Rad1-Rad10 removes damaged sequences, enabling subsequent gap filling and ligation. Acting at mid-steps of repair, the association and dissociation of Rad1-Rad10 with DNA can influence repair efficiency. We show that genotoxin-enhanced Rad1 sumoylation occurs after the nuclease is recruited to lesion sites. A single lysine outside Rad1's nuclease and Rad10-binding domains is sumoylated *in vivo* and *in vitro*. Mutation of this site to arginine abolishes Rad1 sumoylation and impairs Rad1-mediated repair at high doses of DNA damage, but sustains the repair of a single double-stranded break. The timing of Rad1 sumoylation and the phenotype bias toward high lesion loads point to a post-incision role for sumoylation, possibly affecting Rad1 dissociation from DNA. Indeed, biochemical examination shows that sumoylation of Rad1 decreases the complex's affinity for DNA without affecting other protein properties. These findings suggest a model whereby sumoylation of Rad1 promotes its disengagement from DNA after nuclease cleavage, allowing it to efficiently attend to large numbers of DNA lesions.

## INTRODUCTION

Structure-specific nucleases occupy a central position in DNA repair due to their ability to remove a wide range of damaged sequences and resolve joint DNA structures in the genome. Consequently, they greatly influence genome stability and cell survival upon exposure to environmental mutagens and cancer therapeutic drugs. Optimized nuclease function *in vivo* is achieved by multiple layers of regulation. Many structure-specific nucleases interact with and are regulated by other repair factors that help recruit the nucleases to specific DNA lesion sites or stimulate their activities (1–4). In addition, many nucleases are modified post-translationally (4–19). A few detailed studies show that dynamic and reversible protein modifications can alter nuclease activities or protein levels in order to meet specific cell cycle needs for DNA cleavage (11–16). However, the effects of many of these protein modifications on nucleases are currently unknown.

Recent studies have revealed that a large number of DNA repair proteins, including several nucleases, are sumoylated in response to DNA damage in yeast and humans (7–9,20). Although sumoylation as a whole can increase DNA repair capacity (7,20–25), it is unclear how this is achieved at the level of each substrate and what principles underlie SUMO-mediated regulation of DNA repair. A comprehensive understanding of these questions requires detailed studies of sumoylation's effects on each target. Here we look into the role of sumoylation in regulating the Rad1 nuclease in budding yeast. Rad1 forms a heterodimer with Rad10, which is required for Rad1 catalytic activity on branched DNA substrates (26–28). Rad1-Rad10 and their human homologs XPF-ERCC1 can remove several types of DNA lesions, such as those generated by UV radiation, topoisomerase inhibitors and DNA break-inducing agents (1,29). Their important physiological roles are highlighted by the association of XPF-ERCC1 mutations with cancer-prone diseases, including xeroderma pigmentosum, Cockayne syndrome and Fanconi anemia (30–32).

In yeast, Rad1-Rad10 acts in nucleotide excision repair (NER) to remove bulky DNA lesions, such as those induced by UV (29). DNA distortion generated by these lesions is recognized by the NER factors Rad4 and Rad23 (33–35). A pre-incision complex is subsequently formed at lesion sites to unwind the DNA surrounding the lesion, generating a bubble structure (29,36,37). The Rad14 protein of this pre-incision complex recruits Rad1-Rad10 to DNA bubbles via direct physical interaction (38,39). Dual incisions by Rad1-Rad10 and another nuclease, Rad2, at the 5′ and 3′ ends of the bubble, respectively, remove lesion-containing fragments (40,41). This allows subsequent repair synthesis and ligation. Besides involvement in NER, Rad1-Rad10 also acts as a back-up nuclease to remove protein-DNA adducts generated by the Top1 inhibitor camptothecin (CPT) (42,43). Moreover, Rad1-Rad10 functions in single-strand annealing (SSA) repair of double-stranded breaks, where its cleavage of 3′ flaps enables subsequent ligation (44,45). Recruitment and nucleolytic activity of Rad1-Rad10 in SSA are regulated by the lesion-binding factor Saw1 and the scaffolding protein Slx4, respectively (46–48).

Here, we determined that Rad1 is sumoylated on a single lysine and generated an unsumoylatable *rad1* allele. Examining the phenotype of this mutant and the timing of modification *in vivo*, in conjunction with *in vitro* analysis of the sumoylated Rad1 protein, suggests that sumoylation of Rad1 promotes repair efficiency, most likely by enhancing the dissociation of Rad1-Rad10 from DNA after nucleolytic cleavage.

## MATERIALS AND METHODS

### Yeast strains and genetic manipulations

Strains used are listed in Table 1. Standard yeast protocols were used for strain generation, growth, medium preparation and DNA damage sensitivity assays. As *siz1Δ siz2Δ* results in amplification of 2-micron plasmids (49), strains with *siz1Δ siz2Δ* mutations were cured of the plasmid as described (50). Spot assays were performed as described previously (7). Briefly, log phase cells were diluted 10-fold or 3-fold and spotted onto YPD (Yeast extract-Peptone-Dextrose) media with or without CPT, or irradiated with UV. Plates were incubated at 30°C and photographed after 24–72 h.

**Table 1.** Yeast strains used in this study

Strain	Genotype	Source
W1588-4A	*Mat alpha leu2-3,112 ade2-1 can1-100 his3-11,15 ura3-1 trp1-1 RAD5*	R. Rothstein
T581	*Mat a RAD1-TAP::HIS*	This study
X3456*-*2C	*Mat a RAD1-TAP::HIS rad14Δ::KAN*	This study
X3711*-*1B	*RAD1-TAP::HIS rad4Δ::KAN*	This study
X3527*-*9A	*RAD1-TAP::HIS saw1Δ::KAN*	This study
X3539*-*8B	*Mat alpha RAD1-TAP::HIS slx4Δ::KAN*	This study
X3527*-*1A	*Mat a RAD1-TAP::HIS rad14Δ::KAN saw1Δ::KAN*	This study
X3458*-*1A	*Mat alpha RAD1-TAP::HIS rad52Δ::KAN*	This study
X3526*-*13B	*Mat alpha RAD1-TAP::HIS rad14Δ::KAN rad52Δ::KAN*	This study
T1302*-*2	*Mat a rad1-D825A-TAP::HIS*	This study
T908*-*2	*Mat alpha rad1-D869A-TAP::HIS*	This study
X3563*-*1B	*Mat alpha rad1-D869A-TAP::HIS saw1Δ::KAN*	This study
X3541*-*10D	*Mat alpha rad1-D869A-TAP::HIS rad14Δ::KAN*	This study
X3541*-*10A	*Mat alpha rad1-D869A-TAP::HIS rad52Δ::KAN*	This study
X3629*-*20A	*RAD1-TAP::HIS mec1Δ::TRP sml1Δ::HIS*	This study
X3580*-*12A	*Mat alpha RAD1-TAP::HIS siz1Δ::KAN cir0*	This study
X3580*-*4C	*Mat a RAD1-TAP::HIS siz2Δ::URA cir0*	This study
X3201*-*6D	*RAD1-TAP::HIS mms21-11::HIS cir0*	This study
X3580*-*5C	*Mat alpha RAD1-TAP::HIS siz1Δ::KAN siz2Δ::URA cir0*	This study
X3401*-*1C	*Mat alpha rad1Δ::LEU*	This study
X3840*-*6B	*rad1-K32R-TAP::HIS*	This study
X5692*-*7A	*RAD1-TAP::HIS apn2Δ::HIS*	This study
X3919*-*2D	*rad1-K32R-TAP::HIS apn2Δ::HIS*	This study
X6004*-*1B	*rad1Δ::LEU apn2Δ::HIS*	This study
X4373*-*17A	*RAD1-TAP::HIS tdp1Δ::KAN*	This study
X3916*-*1C	*rad1-K32R-TAP::HIS tdp1Δ::KAN*	This study
X3404*-*2D	*rad1Δ::LEU tdp1Δ::HIS*	This study
X3727*-*1A	*RAD1-TAP::HIS RAD10-13Myc::KAN*	This study
X3840*-*5C	*rad1-K32R-TAP::HIS RAD10-13Myc::KAN*	This study
X3729*-*1A	*RAD1-TAP::HIS RAD14-13Myc::KAN*	This study
X4491*-*5A	*rad1-K32R-TAP::HIS RAD14-13Myc::KAN*	This study
X4965*-*2C	*RAD1-TAP::HIS SAW1-FLAG::KAN*	This study
X4966*-*9A	*rad1-K32R-TAP::HIS SAW1-FLAG::KAN*	This study
X4489*-*1C	*RAD1-TAP::HIS SLX4-3HA::KAN*	This study
X4490*-*1B	*rad1-K32R-TAP::HIS SLX4-3HA::KAN*	This study
X3728*-*1A	*RAD10-13Myc::KAN*	This study
X4491*-*3B	*RAD14-13Myc::KAN*	This study
X4965*-*2B	*SAW1-FLAG::KAN*	This study
X4489*-*1B	*SLX4-3HA::KAN*	This study
SLY5151	EAY1141 *ho HML mat::leu2::hisG hmrΔ3 leu2-3,112 ura3-52 trp1 THR4-ura3-A(205bp)-HOcs-URA3-A ade3::GAL10-HO::NAT*	This study
SLY5136	SLY5151 *rad1-K32R-3HA::HYG*	This study

Yeast strains are listed in Table 1. Strains in this study except SSA strains are derivatives of W1588–4C, a *RAD5* derivative of W303.

Thomas, B.J. and Rothstein, R. (1989) Elevated recombination rates in transcriptionally active DNA. *Cell*, **56**, 619–630*.*

Sugawara, N., Goldfarb, T., Studamire, B., Alani, E. and Haber, J.E. (2004) Heteroduplex rejection during SSA requires Sgs1 helicase and mismatch repair proteins Msh2 and Msh6 but not Pms1. *Proc. Natl. Acad. Sci. U. S. A.*, **101**, 9315–9320.

### Detection of sumoylated proteins and immunoprecipitation

**Figure 1.**
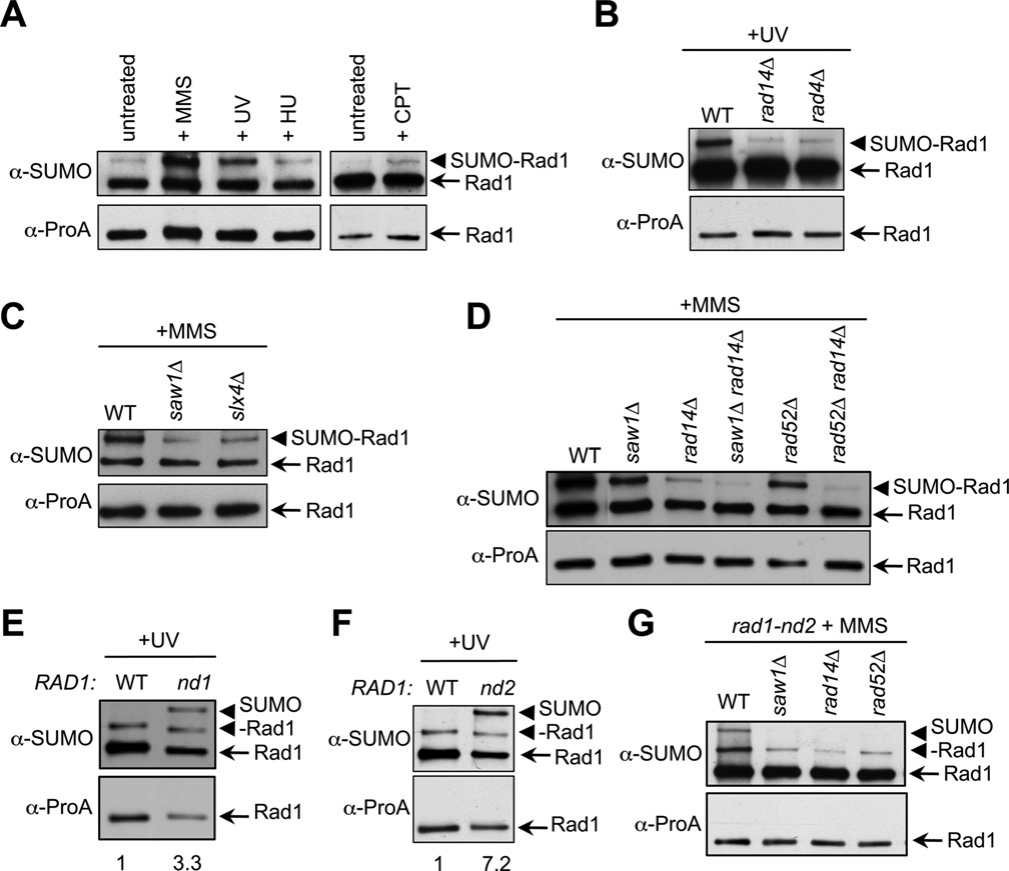
DNA damage-induced sumoylation of Rad1 occurs upon recruitment to lesion sites. **(A)** Rad1 sumoylation is induced by UV, MMS and CPT, but not HU treatment. TAP-tagged Rad1 was immunoprecipitated from yeast strains untreated or treated with 100 J/m^2^ UV, 50 ug/ml CPT, 0.2 M HU or 0.2% MMS and was western blotted for SUMO (top) and Protein A (bottom). Note that in the SUMO blot, the lower band representing the unmodified form arises from interaction of the nonspecific region of the antibody with the Protein A (ProA) portion of TAP (7). **(B–D)** Rad1 sumoylation induction requires upstream NER and SSA factors. Cells containing Rad1-TAP and indicated mutations were treated with 200 J/m^2^ UV (B) or 0.3% MMS (C–D) and examined as in (A). **(E–G)** Hypersumoylation of nuclease dead Rad1-D825A (−nd1) and Rad1-D869A (−nd2) depends on upstream NER and SSA factors. Cells containing indicated mutations were examined as in (B–C). In (E) and (F), the relative ratios of modified to unmodified forms were quantified using the band intensities from the SUMO blot and are shown below. Different intensities of unmodified and sumoylated Rad1 bands on different blots are due to variable antibody quality and exposure time.

These were performed as described previously (7). In brief, cells were lysed by bead beating under denaturing conditions and TAP-tagged proteins were immunoprecipitated using immunoglobulin G (IgG)-Sepharose. These were then washed and eluted with loading dye, followed by sodium dodecylsulphate-polyacrylamide gel electrophoresis (SDS-PAGE) and western blotting with antibodies against SUMO (22) and the protein A portion of the TAP tag (Sigma-Aldrich). Damage-induced sumoylation was assayed by subjecting log-phase cells to 0.03–0.3% methylmethane sulfonate (MMS, Sigma-Aldrich), 50 ug/ml CPT (Sigma-Aldrich), or 0.2 M hydroxyurea (HU, US Biologicals) for 2 h, or UV irradiation using UV Stratalinker 1800 (Stratagene). For Figure 1A, UV and MMS doses were chosen based on comparable survival posttreatment as shown in Supplementary Figure S1A–B. Co-immunoprecipitation experiments were performed as described previously (51).

### SSA assays

Chromosomal and plasmid-based SSA assays were performed as described (46). In brief, for chromosomal assay, log-phase cells grown in YP-Glycerol were plated on either YPD or YP-Galactose media. Only the latter induces HO expression, and thus double -strand break formation and SSA repair. Colonies were counted after incubation at 30°C for 3–4 days. SSA survival quantification was done by dividing the number of viable colonies on YP-galactose plates by that on YPD and multiplying this factor by 100. For plasmid-based assay, cells were transformed with either *Bsu*36I- or mock-digested pNSU208 plasmid, plated on media lacking leucine and incubated at 30°C for 3–4 days. Percent survival estimating SSA efficiency was calculated by dividing number of viable colonies of the *Bsu*36I-digested transformants by those of mock-digested ones.

### Rad1-Rad10 protein expression, purification and gel filtration

The *Escherichia coli* strain Rosetta(DE3)pLysS was transformed with a bicistronic plasmid (gift from Dr. Steve Brill (52)) expressing (His)_6_-Rad1 and Rad10, or (His)_6_-Rad1-K32R and Rad10. The Rad1-K32R mutant was generated using site-directed mutagenesis. Protein expression was induced by 0.1 mM IPTG (Isopropyl β-D-1-thiogalactopyranoside) at 16°C overnight. A total of 9 g of cell paste was sonicated in 40 ml breakage buffer (50 mM Tris-HCl, pH 7.5, 10% sucrose, 2 mM ethylenediaminetetraacetic acid (EDTA), 200 mM KCl, 0.01% NP40, 1 mM β-mercaptoethanol and protease inhibitor cocktail containing pepstatin, aprotinin, benzamidine, chymostatin and leupeptin). The lysate was clarified by centrifugation (100k g, 4°C, 1 h), and the supernatant was incubated with 1 ml of HIS-Select nickel affinity gel (Sigma) for 1 h at 4°C. The beads were washed with 12 ml of buffer K (20 mM K_2_HPO_4_, 10% glycerol, 0.5 mM EDTA) containing 150 mM KCl. The bound proteins were eluted with buffer containing 50 mM KCl and imidazole (from 50 to 1000 mM). Pooled fractions containing Rad1-Rad10 (150–500 mM imidazole) were applied to a 1-ml Heparin column (GE Healthcare) followed by elution using 8 ml gradient of 275–1000 mM KCl in buffer K. The peak fractions of Rad1-Rad10 at ∼500 mM KCl elution were pooled, loaded onto a 0.5-ml MonoQ column (GE Healthcare) and eluted using a 5-ml gradient of 275–1000 mM KCl in buffer K. The Rad1-Rad10 fractions were concentrated to 400 μl in a VivaSpin-2 concentrator, and fractionated on a 23-ml Sephacryl S400 column (GE Healthcare) in K buffer containing 300 mM KCl. Peak Rad1-Rad10 fractions were concentrated to 2 μg/μl. To determine the oligomeric status of Rad1-Rad10, a 23 ml Sephacryl S400 column was eluted with buffer K containing 300 mM KCl (0.11 ml/min flow rate), and 0.35 ml fractions were collected. The indicated fractions were separated by SDS-PAGE and detected by western blotting using α-Rad1 antibody (Santa Cruz).

### *In vitro* sumoylation assay

Purification of GST-Aos1/Uba2, His-Ubc9, His-Flag-Smt3, His-Flag-Smt3-KR, His-Siz1 (1–465) and Siz2 and the sumoylation assay were performed as previously (53). In brief, the 10 μl reaction contained 150 nM Aos1/Uba2, 0.5 μM Ubc9, 4.3 μM Smt3 or Smt3-KR, 0.4 μM Rad1-Rad10, 1 mM adenosine triphosphate (ATP) and buffer S1 (100 mM Tris-HCl pH 7.5, 10 mM MgCl_2_) (53). In the indicated cases, 10–100 nM Siz1 or 10–100 nM Siz2 was added to the reaction.

### DNA substrates

All substrates were prepared as described (54). Synthetic oligonucleotides were purchased from VBC Biotech; sequences are available upon request.

### Electrophoretic mobility shift assay (EMSA)

Rad1-Rad10 was incubated with fluorescently labeled DNA substrates (4 nM) in 10 μl buffer D (40 mM Tris-HCl, pH 7.5, 50 mM KCl, 1 mM DTT and 100 μg/ml bovine serum albumin) at 37°C for 10 min. The reactions were stopped by adding loading buffer (60% glycerol, 10 mM Tris, pH 7.4 and 60 mM EDTA). Samples were separated on native polyacrylamide gel (10%) in 0.5×TBE buffer (40 mM Tris-HCl, 20 mM boric acid, 2 mM EDTA, pH 7.5). DNA was visualized by a scanner FLA9000 Starion (Fujifilm) and quantified using MultiGauge software (Fujifilm).

### Nuclease assay

Rad1-Rad10 was incubated with fluorescently labeled DNA substrate (4 nM) in 10 μl buffer R (50 mM Tris-HCl, pH 7.5, 1 mM MgCl_2_, 1 mM DTT and 100 μg/ml bovine serum albumin). The reaction mixtures were incubated at 30°C for 30 min and deproteinized by adding 0.1% SDS and 500 μg/ml of proteinase K for 5 min at 37°C. Samples were resolved on a 10% native polyacrylamide gel in TBE buffer. The fluorescent DNA species were visualized as in EMSA.

## RESULTS

### Rad1 sumoylation is enhanced by DNA damaging agents and requires Rad1 recruitment to DNA lesions

Recent proteomic and biochemical screens have identified Rad1 as one of the sumoylation targets during the DNA damage response (7,8,20). We confirmed Rad1 sumoylation in our W303 strain background using endogenously expressed and functional TAP-tagged Rad1 (Figure 1A, and data not shown). The sumoylated form of Rad1 was detected as a single band above the unmodified protein band on western blots when immunoprecipitated Rad1 was examined with anti-SUMO antibody (Figure 1A). Rad1 sumoylation was observed under normal growth conditions and an increase was seen upon exposure to UV, the alkylation agent MMS and the Top1 poison CPT (Figure 1A and Supplementary Figure S1C). Treatment with the ribonucleotide reductase inhibitor HU that is not known to require Rad1-mediated repair had no effect (Figure 1A). We note that even upon UV, MMS and CPT exposure, only a small proportion of Rad1 was sumoylated, as the modified Rad1 band was barely detectable on blots probed with anti-tag antibody after short exposures. We conclude that a small fraction of Rad1 is mono-sumoylated and this modification is enhanced by UV, MMS and CPT treatment.

The low level of Rad1 sumoylation and its DNA damage inducibility raised the possibility that Rad1 is sumoylated only in specific situations, such as when the protein is actively engaged in DNA repair. We addressed this possibility by examining whether Rad1 sumoylation depended on its recruitment to lesions. Under UV treatment, Rad14 recruits Rad1-Rad10 to DNA lesions by physical interaction (38). We found that *rad14Δ* greatly reduced the level of Rad1 sumoylation (Figure 1B). We also examined whether Saw1, a Rad1 recruitment factor in SSA (46,47), influences its sumoylation. Since SSA mutants are most sensitive to MMS treatment, we conducted the test in MMS and found that *saw1Δ* also reduced Rad1 sumoylation (Figure 1C). Residual sumoylation in *rad14Δ* and *saw1Δ* cells likely reflects Rad1 recruitment to DNA lesions by the remaining factor. Indeed, *rad14Δ saw1Δ* double mutants exhibited further reduction of Rad1 sumoylation than either single mutant (Figure 1D).

As Rad14 and Saw1 both recruit and form complexes with Rad1-Rad10, the observed Rad1 sumoylation decrease could be due to either impaired recruitment or impaired complex formation in their absence. To discern if Rad1-Rad10 recruitment *per se* is important for Rad1 sumoylation, we examined more upstream NER and SSA proteins that are necessary for Rad1-Rad10 recruitment but do not physically interact with the nuclease. In NER, Rad1 recruitment to UV lesions requires the lesion recognition factor Rad4, which does not bind to Rad1-Rad10 (55). We found that *rad4Δ*, like *rad14Δ*, reduced Rad1 sumoylation (Figure 1B), suggesting that it is the presence of Rad1 at DNA lesions that is essential for its sumoylation. Similarly, in mutants of the SSA factor Rad52, which is required for formation of 3′ flaps and Rad1 recruitment but does not physically interact with Rad1 (44), we also detected reduced Rad1 sumoylation, as in *saw1Δ* cells (Figure 1D). Also as in *saw1Δ* cells, the residual sumoylation in *rad52Δ* was largely dependent on Rad14, as the *rad52Δ rad14Δ* double mutant showed a further decrease in Rad1 sumoylation (Figure 1D), consistent with both NER and SSA contributing to the repair of MMS lesions. Taken together, the reduction in Rad1 sumoylation in mutants lacking two NER proteins, Rad4 and Rad14, and two SSA proteins, Saw1 and Rad52, suggests that the initiation of repair and subsequent recruitment of Rad1-Rad10 to DNA lesions are required for Rad1 sumoylation.

### Mutations blocking Rad1-mediated cleavage affect Rad1 sumoylation

The above results suggest that Rad1 sumoylation occurs after it is recruited to DNA lesions. Next we addressed whether DNA lesion recruitment is sufficient to induce Rad1 sumoylation, using the *slx4Δ* mutant. Slx4 is required for Rad1 nucleolytic cleavage but not its recruitment to 3′ flaps during SSA (46,47,56). In *slx4Δ* cells, Rad1 is recruited to SSA lesions but cannot initiate cleavage (46,47). We found that *slx4Δ* reduced Rad1 sumoylation similarly to *saw1Δ* (Figure 1C). This effect suggests that Rad1 sumoylation requires an Slx4-dependent step after DNA lesion association.

To test if nucleolytic cleavage is required for sumoylation, we used two nuclease dead Rad1 mutants. The D825A mutant (Rad1-nd1) lacks a conserved catalytic residue and is trapped on DNA (46), and the D869A mutant (Rad1-nd2) corresponds to D720A in XPF that shows no nuclease activity *in vitro* (42,57). Interestingly, both catalytic site mutations increased Rad1 sumoylation, as relative levels of SUMO-Rad1 over unmodified forms were higher in *rad1-nd1* and *–nd2* cells than in wild-type (Figure 1E and F. Moreover, both mutants showed additional sumoylated Rad1 species (Figure 1E and F. The increased sumoylation still depended on Rad1 recruitment to DNA lesions as it was reduced in mutants of upstream NER and SSA factors, including Rad14, Saw1 and Rad52 (Figure 1G). This result, in conjunction with our other data, suggests that Rad1 sumoylation occurs at lesion sites and requires an Slx4-dependent step but not Rad1-Rad10 nucleolytic activity.

### Sumoylation of Rad1 after DNA damage is Mec1-independent and requires the Siz ligases

**Figure 2.**
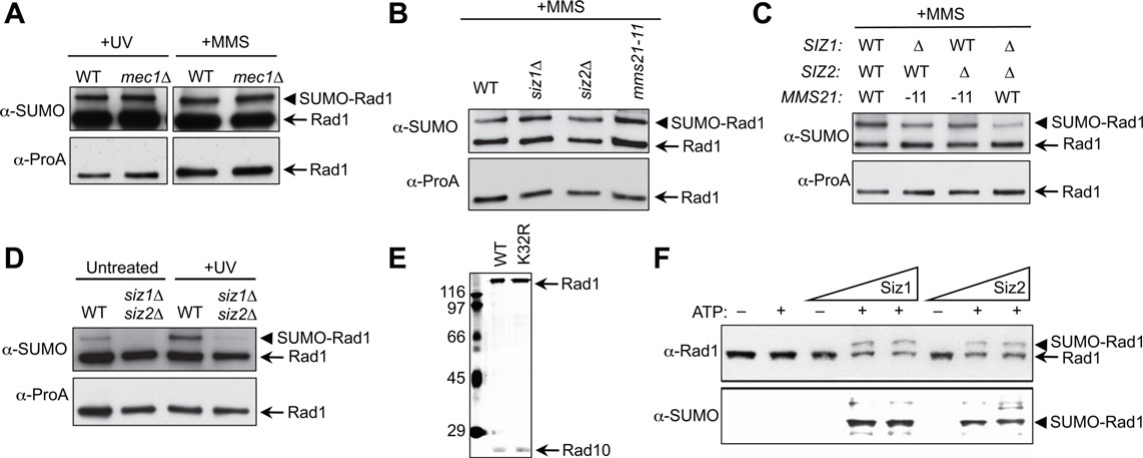
Sumoylation induction of Rad1 is dependent on Siz1 and Siz2 but not Mec1. **(A)** Rad1 sumoylation upon MMS and UV treatment does not require Mec1. Rad1-TAP from wild-type or *mec1Δ* strains was assayed as in Figure 1B and C. **(B–D)** Siz1 and Siz2 primarily contribute to Rad1 sumoylation *in vivo*. Cells containing Rad1-TAP and indicated mutations were assayed as in Figure 1B and C**. (E)** Coomassie stain showing purified His-tagged recombinant Rad1-Rad10 and Rad1-K32R-Rad10 complexes. **(F)** Rad1 sumoylation is stimulated by the Siz1 and Siz2 ligases *in vitro*. Reactions were incubated with increasing concentrations of Siz enzymes (10–100 nM) for 1 h at 30°C and analyzed by 10% SDS–PAGE, followed by western blotting with antibodies against Rad1 (top) and SUMO (bottom).

Several aspects of DNA repair are regulated by the DNA damage checkpoint (58,59). However, we have previously shown that the DNA damage-induced sumoylation of numerous proteins is checkpoint-independent, indicating that this sumoylation response is largely separable from the phosphorylation-based checkpoint pathway (7,60). To understand if Rad1 sumoylation follows this rule or is an exception, we analyzed its sumoylation in the absence of the main checkpoint kinase Mec1. As shown in Figure 2A, the level of Rad1 sumoylation was the same in *mec1Δ* cells as in wild-type after treatment with UV or MMS. Thus, we conclude that damage-induced sumoylation of Rad1, as for many other repair proteins, is not dependent on Mec1.

Yeast, like most organisms, contains only one SUMO E2, but multiple mitotic SUMO E3 ligases. Yeast E3 ligases include the homologous Siz1 and Siz2 proteins, and the more distant Mms21. The Siz enzymes, and sometimes Mms21, show redundancy in sumoylation of various substrates (61,62). To understand whether Rad1 sumoylation is dependent on a particular E3 or can be carried out by redundant E3s, we examined single and double E3 mutants *in vivo*. We found that none of the single E3 mutants affected Rad1 sumoylation level (Figure 2B). For combinatorial E3 mutants, only the *siz1Δ siz2Δ* double mutant significantly decreased Rad1 sumoylation, while combining the *siz* single mutations with *mms21–11*, which lacks the Mms21 ligase domain, resulted in smaller decreases (Figure 2C and D. The redundancy of the two Siz proteins is consistent with the observation that the *siz1Δ siz2Δ* double mutant, but neither single mutant, is sensitive to UV and MMS ((7,20) and data not shown). Thus, the three E3s are redundant in sumoylating Rad1 *in vivo*, with Siz1 and Siz2 playing prominent roles.

To test Siz-dependent sumoylation *in vitro*, we purified recombinant Rad1-Rad10, as well as SUMO machinery proteins, including SUMO (Smt3), E1 (Aos1/Uba2), E2 (Ubc9) and the Siz E3s, as previously described ((53), Figure 2E and data not shown). As shown in Figure 2F, Siz1 or Siz2 can efficiently sumoylate Rad1 in the presence of ATP. In both cases, a single Rad1 sumoylation band was detected by antibodies against Rad1 or SUMO on western blots. These observations are consistent with our *in vivo* findings, indicating that Rad1 can be sumoylated by both Siz ligases. We note that *in vitro* sumoylation requirements are more relaxed than *in vivo*, likely due to the high concentration of sumoylation machinery components (63–65). Consistent with this, the presence of DNA and/or Saw1 did not appear to affect Rad1 sumoylation (data not shown).

### Rad1 sumoylation occurs on a single lysine *in vivo* and *in vitro*

**Figure 3.**
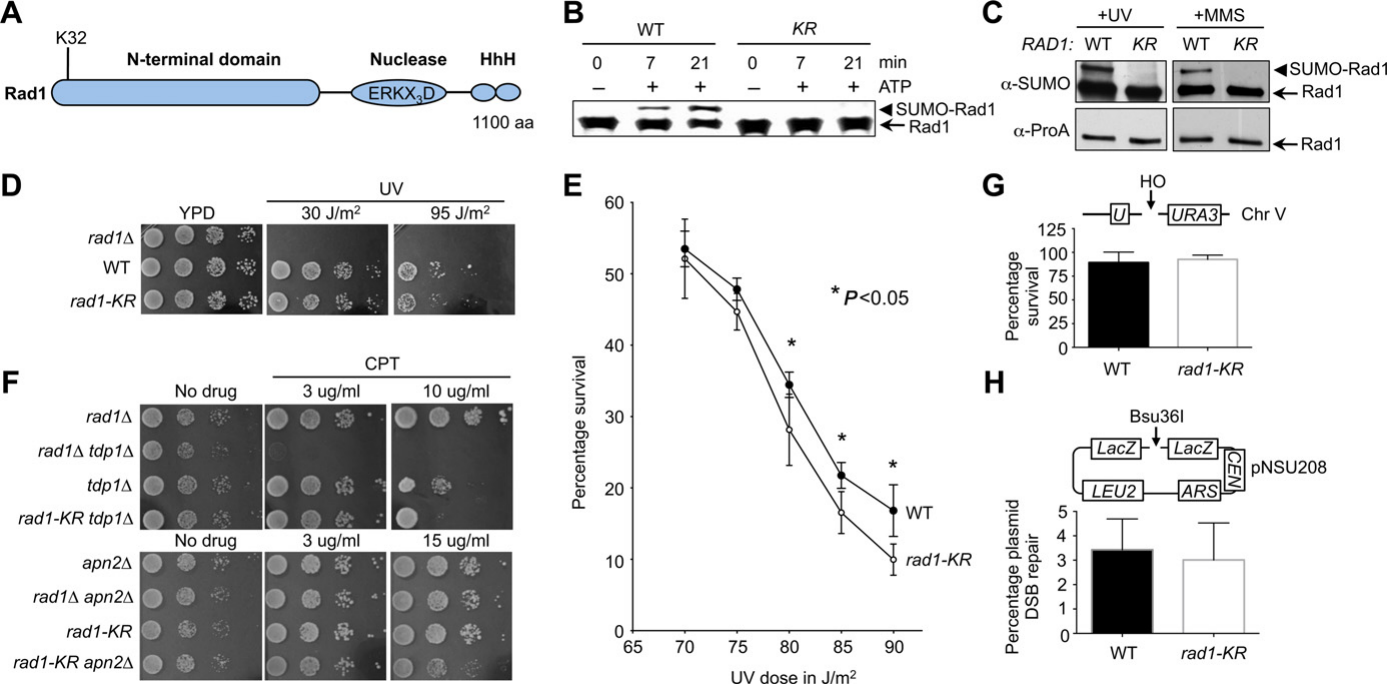
Rad1 is monosumoylated at K32 and its sumoylation contributes to UV and CPT repair. **(A)** Schematic depicting Rad1 domains and sumoylation site. HhH denotes helix–hairpin–helix. **(B)** Rad1-K32R is not sumoylated *in vitro*. Proteins purified as in Figure 2E were assayed for sumoylation as in Figure 2F. **(C)***rad1-K32R* abolishes Rad1 sumoylation *in vivo*. Rad1-TAP from wild-type or *rad1-K32R* cells was assayed as in Figure 1B and C. **(D and E)***rad1-K32R* is sensitive to UV at high doses. Spot assay showing 3-fold serial dilutions of yeast strains either untreated or treated with indicated UV doses (D). Survival curves after exposure to the indicated UV doses is presented based on 5 independent trials; averages and standard deviations are shown (E). Asterisks denote statistically significant differences. **(F)***rad1-K32R* sensitizes *tdp1Δ* and *apn2Δ* to CPT. Spot assay showing 10-fold serial dilutions of yeast strains on indicated media. **(G and H)***rad1-K32R* is not defective in chromosomal (G) and plasmid-based (H) SSA assays. Assays measuring SSA between 205 bp *ura3* repeats on yeast chromosome V (G) or plasmid pNSU208 carrying 240 bp of directly repeated LacZ sequences (H). Survival of wild-type and *rad1-K32R* derivative upon HO induction is shown.

Despite the less stringent requirements for Rad1 sumoylation *in vitro*, the similar sumoylation patterns and the same Siz E3 dependence *in vitro* and *in vivo* suggested that *in vitro* sumoylated Rad1 can be informative for determining the sumoylation site using mass spectrometry. This experiment identified lysine 32 as the sumoylation site with high confidence (*P* < 0.05, Supplementary Figure S2). K32 lies within a sumoylation consensus motif (ψKxE/D, where ψ is a hydrophobic amino acid, (66,67)). This residue in the N-terminal region of the protein is far from Rad1's nuclease domain and the helix–hairpin–helix (HhH) domain involved in interaction with Rad10 and DNA (Figure 3A). The Rad1 N-terminal domain has not been studied in detail, though the same region in the human homolog, XPF, contributes to DNA interaction (68). Thus, the Rad1 sumoylation site localizes to a region likely involved in DNA binding.

To verify that K32 is indeed the *bona fide* sumoylated lysine, we first purified a recombinant K32R mutant of the Rad1 protein and subjected it to *in vitro* sumoylation. As shown in Figure 3B, wild-type Rad1, but not the K32R mutant, can be sumoylated in the presence of ATP. We then tested if K32R also abolishes sumoylation *in vivo*, by introducing the *K32R* mutation at the endogenous *RAD1* locus and assaying sumoylation. *rad1-K32R* eliminated Rad1 sumoylation under both UV and MMS conditions (Figure 3C), confirming that K32 is the Rad1 sumoylation site *in vivo*.

### The *rad1-K32R* mutant shows sensitivity to high doses of UV and CPT, but is proficient for repair of a single DNA break

Next, we examined how lack of Rad1 sumoylation affects cell survival in the presence of genotoxins. We first queried the UV sensitivity of cells expressing the nonsumoylatable *rad1-K32R* allele using both spotting and plate-out assays. In both assays, blocking Rad1 sumoylation decreased resistance to high doses of UV (Figure 3D and E, with an estimated reduction of ∼20% in the survival of *rad1-K32R* cells compared to wild-type cells (*P* < 0.05).

We did not detect increased sensitivity of *rad1-K32R* to other types of DNA damaging agents, including CPT, MMS and HU (data not shown). As Rad1 acts in a back-up pathway for CPT repair and its sumoylation is induced by CPT treatment ((42); Figure 1A), we examined if *rad1-K32R* affects resistance to this drug when other proteins involved in this repair are absent. Like *rad1Δ* but to a less degree, *rad1-K32R* sensitized cells lacking Tdp1, a phosphodiesterase that processes the majority of CPT lesions ((42,69); Figure 3F). As reported previously, *rad1Δ* did not sensitize cells lacking Apn2, an endonuclease that functions in base excision and CPT repair, as well as in processing blocked DNA ends with Rad1 (43,70,71); unexpectedly, *rad1-K32R* showed sensitization (Figure 3F). It is possible that unsumoylated Rad1 prevents downstream or other repair in the absence of Apn2 (see Discussion). In both *tdp1Δ* and *apn2Δ* backgrounds, *rad1-K32R* sensitization was observed only at high CPT doses (Figure 3F).

The manifestation of *rad1-K32R*'s effects at high drug doses suggests that sumoylation influences aspects of Rad1 function that become more critical when the lesion burden is large. To test this idea, we examined how *rad1-K32R* affects the repair of a single double-strand break via SSA. Rad1-Rad10 cleaves at the ds-ssDNA junction in this process as in NER and CPT repair. If the above idea were correct, one would expect that *rad1-K32R* is proficient for repairing a single break. We used two well-established SSA assays where a double-strand break is generated either on a plasmid or at a chromosomal locus by the HO endonuclease (46). As the HO cut site is flanked by two complementary sequences, repair of the break is mediated by SSA. We found that *rad1-K32R* was proficient for SSA repair of both plasmid and chromosomal breaks, based on survival rates after HO induction (Figure 3G-H). Taken together, the phenotype of *rad1-K32R* supports the notion that Rad1 sumoylation becomes more important when large numbers of DNA lesions need to be repaired.

### Lack of Rad1 sumoylation affects neither its protein interactions nor its nuclease activity

**Figure 4.**
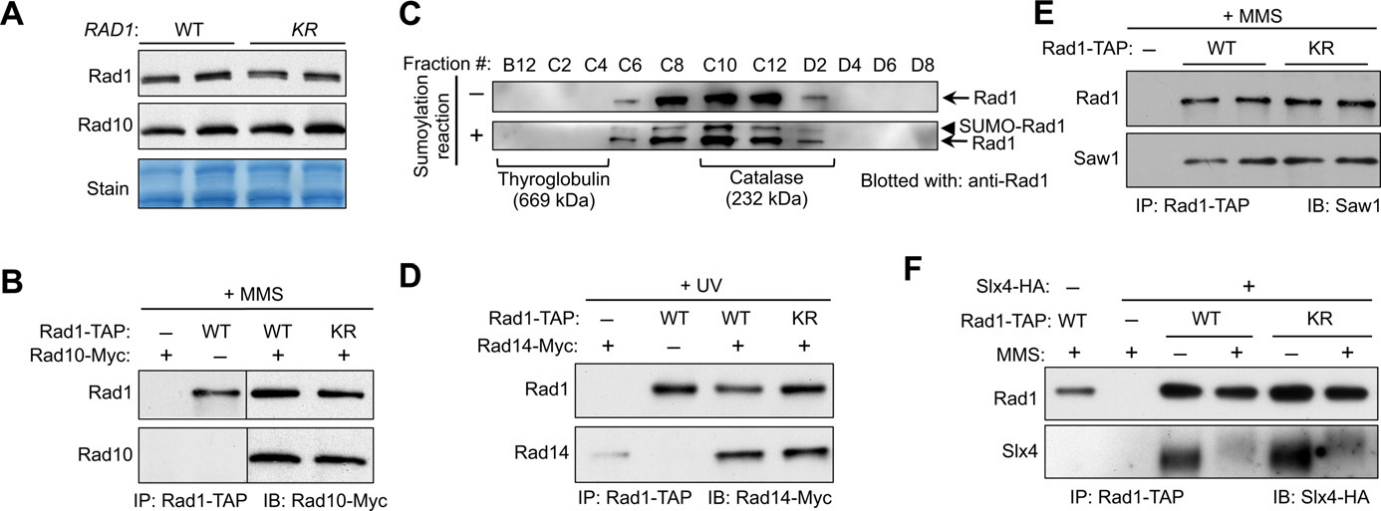
Rad1 sumoylation affects neither Rad1 protein levels nor its protein–protein interactions. **(A)***rad1-K32R* does not affect Rad1 or Rad10 protein levels. Rad1-TAP and Rad10-Myc protein levels in crude extracts from wild-type and *rad1-K32R* mutant cells were assayed by western blotting with antibodies against corresponding tags. **(B, D–F)** Rad1-K32R is proficient for interaction with Rad10, Rad14, Saw1 and Slx4 *in vivo*. Extracts from cells containing indicated tagged proteins treated with MMS or UV were immunoprecipitated with IgG-Sepharose to pull down Rad1-TAP, and probed by western blot using antibodies against either the corresponding tag or Saw1. Note that in (D), the faint Rad14 band present in the untagged Rad1 sample (first lane) is due to nonspecific binding of Rad14-Myc to the resin. The Rad14-Rad1 interaction is manifested in the increased amount of Rad14 pulled down from Rad1-TAP compared with untagged Rad1. **(C)** Sumoylated Rad1 has the same gel filtration profile as unmodified protein. Recombinant Rad1-Rad10 was assayed by gel filtration before and after being subjected to sumoylation reaction. Molecular markers are indicated below the gel filtration profiles.

To gain a molecular understanding of the effect of Rad1 sumoylation and of the reason underlying the *rad1-K32R* phenotype, we examined Rad1 protein properties in several ways. First, *rad1-K32R* did not affect Rad1 protein levels under normal and genotoxin conditions (Figure 4A; data not shown). Second, Rad1-K32R behaved similarly to wild-type Rad1 in DNA binding assays (Supplementary Figure S3A and B). Third, *rad1-K32R* did not affect interaction with Rad10, as wild-type and mutant Rad1 proteins exhibited similar levels of Rad10 association *in vitro* and *in vivo* (Figures 2E and 4B). Additionally, the gel filtration profiles of unmodified and sumoylated Rad1 in complex with Rad10 were similar, with sumoylated Rad1 being eluted in the same fractions as the unmodified protein (Figure 4C). Thus, we conclude that the sumoylation status of Rad1 does not affect Rad10 interaction. We note that the gel filtration profiles of Rad1-Rad10 proteins are consistent with a dimer of heterodimers with an apparent molecular mass of 300 kDa (Figure 4C), though further work is necessary to gain a detailed understanding of this potential oligomerization.

Next, we examined Rad1 protein interactions important for its recruitment and/or nuclease activity. First, we tested Rad1 interaction with Rad14 after UV treatment, as NER is critical for UV repair. No difference was seen between Rad1 and Rad1-K32R in their ability to pull down Rad14 protein after UV treatment (Figure 4D). Similar results were obtained when wild-type Rad1 and the K32R mutant were assessed for Saw1 or Slx4 interactions (Figure 4E and F. Note that the Slx4 band shift after MMS treatment was seen as previously reported and is due to phosphorylation of the protein (72). The reason for lower levels of Slx4 pulled down after MMS treatment is unclear, though it could be due to either reduced Slx4-Rad1 interaction or less efficient detection. Nevertheless, the Rad1-K32R mutant behaved similarly to the wild-type protein in its ability to interact with Slx4 in this assay (Figure 4F). Taken together, we conclude that Rad1-K32R is proficient for interactions with Rad14, Saw1 and Slx4, suggesting that sumoylation of Rad1 does not cause major changes in protein–protein interactions.

**Figure 5.**
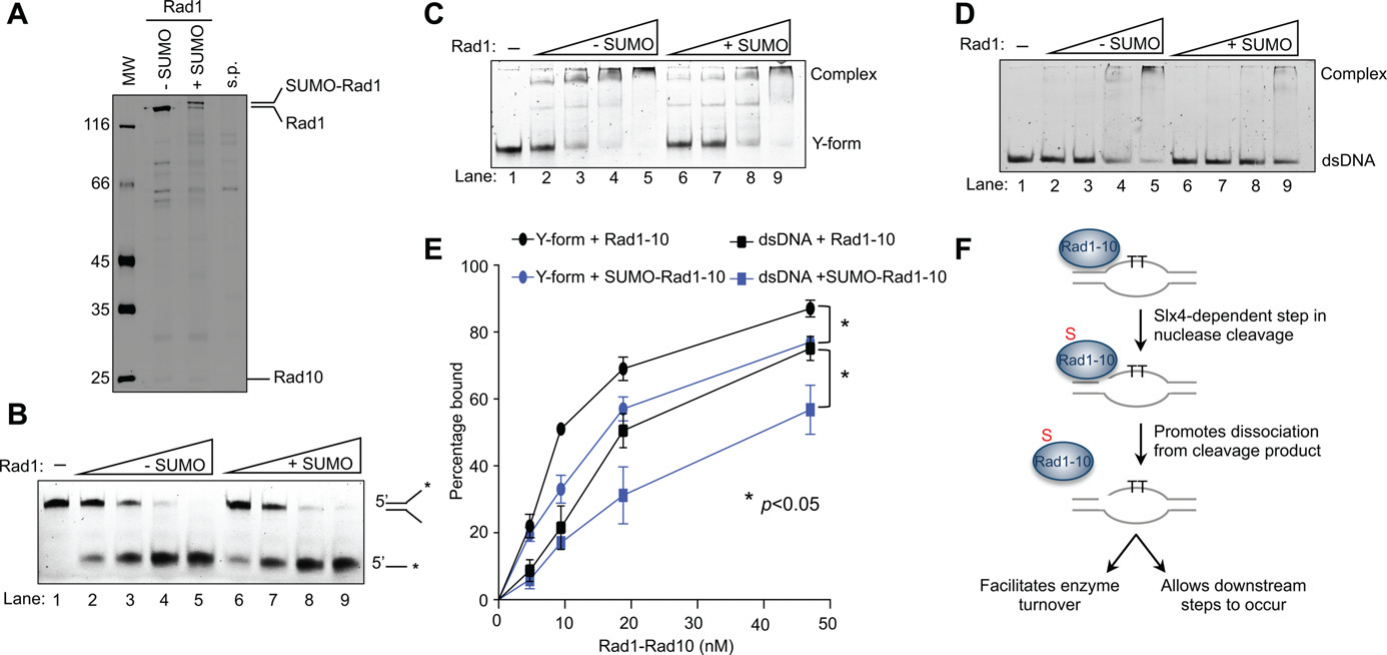
Rad1 sumoylation leads to decreased affinity for DNA but does not affect nuclease activity. **(A)** Rad1 sumoylation reaction *in vitro*. s.p. denotes sumoylation reaction mixture without Rad1-Rad10. **(B)** Sumoylated Rad1 complexed with Rad10 has the same nuclease activity on Y-forms as the unmodified complex. Increasing concentrations of sumoylated (lanes 6–9) and nonsumoylated (lanes 2–5) Rad1-Rad10 proteins (0.06–1.2 nM) were incubated with Y-form DNA (4 nM) and assayed as described in Methods. **(C–E)** Sumoylated Rad1 complexed with Rad10 exhibit decreased affinity for Y-forms (C) and dsDNA (D). Increasing concentrations of sumoylated (lanes 6–9) and nonsumoylated (lanes 2–5) Rad1–10 proteins (5–50 nM) were tested. Quantification of several binding trials is shown in (E); Rad1–10 denotes Rad1-Rad10. Asterisks indicate statistically significant differences. **(F)** Model: Sumoylation of Rad1 promotes dissociation of the Rad1-Rad10 complex from the cleavage product. This effect likely occurs in NER (depicted), CPT and SSA repair (not shown). Details in Discussion.Discussion.

To test the effect of sumoylation on Rad1 nuclease activity, we compared the activity of equal amounts of wild-type Rad1-Rad10 with the K32R variant complex on Y-form substrates. The fluorescently labeled DNA cleavage product migrated faster than the reactant on gels and the mutant complex exhibited activity similar to its wild-type counterpart (Supplementary Figure S3C). We also optimized our *in vitro* sumoylation system such that ∼80% of Rad1 was sumoylated (Figure 5A). When we compared equal amounts of sumoylated and unmodified Rad1 in these cleavage assays, again, no difference was detected (Figure 5B). As control reactions that contain only the sumoylation reaction proteins did not show any nuclease activity (Supplementary Figure S4A), the cleaved product was produced by sumoylated Rad1, rather than other components of the sumoylation reaction. We note that the presence of 20% unmodified Rad1 may exclude the detection of small effects of sumoylation. Together, these results show that neither the lack of Rad1 sumoylation nor using a protein prep containing predominantly sumoylated Rad1 affects nuclease activity.

### Sumoylation of Rad1 decreases its affinity for dsDNA and Y-forms

Our *in vivo* data suggest that Rad1 sumoylation occurs after DNA lesion recruitment and that the modification becomes more important when dealing with large numbers of DNA lesions. Both observations suggest that sumoylation may aid the release of Rad1 from the product after nuclease cleavage. Thus, we examined whether sumoylation of Rad1 favors DNA dissociation.

We tested the interaction of unmodified and sumoylated Rad1-Rad10 with both Y-form DNA and dsDNA, which mimic the substrate and product of the nucleolysis reaction, respectively. We found that unmodified Rad1-Rad10 binds to both dsDNA and Y-forms (Figure 5C and D. Although Rad1-Rad10 is thought to be released from DNA after cleavage simply by intrinsic differences in the enzyme's affinity for different forms of DNA, we found that Rad1-Rad10 interaction with dsDNA is only slightly weaker than that with Y-forms. This result suggests that additional regulation exists to enable efficient dissociation of Rad1-Rad10 from dsDNA. Consistent with this, the excised oligomer in NER is released at a faster rate *in vivo* than *in vitro* (73), implying the existence of regulatory mechanisms specifically for achieving shorter turnaround times in NER *in vivo*.

When sumoylated Rad1 obtained as above was assayed, we detected a reproducible decrease in affinity for both Y-forms and dsDNA, compared with unmodified Rad1 (Figure 5C–E). This is not due to the sumoylation reaction components, as this mixture did not show DNA association (Supplementary Figure S4B and C). In addition, the effect of sumoylated Rad1 is not mimicked by Rad1-K32R-Rad10 in combination with free SUMO (Supplementary Figure S4D and E), suggesting that only the Rad1-conjugated form of SUMO exerts the observed effect on DNA association. We also found that sumoylated Rad1 exhibited less binding to 5′ overhang, but not ssDNA, compared to unmodified Rad1 (Supplementary Figure S4F–H). It is conceivable that the observed sumoylation-mediated reductions in affinity for the product of the cleavage reaction enable efficient disengagement of Rad1-Rad10 from DNA postcleavage.

## DISCUSSION

The Rad1-Rad10 nuclease and homologs are involved in multiple DNA repair pathways and are tightly regulated at the levels of recruitment and activation. Despite the advances in understanding the regulation of this important type of nucleases, the full picture of how they can efficiently attend to large numbers of DNA lesions via multiple pathways has been unclear. Here, we show that budding yeast Rad1 is modified by SUMO after recruitment to lesion sites. Lack of Rad1 sumoylation leads to cellular sensitivity to high doses of DNA damage. These results suggest that sumoylation may affect Rad1 function by enabling efficient recycling of the enzyme. Our biochemical studies provide direct evidence that sumoylation of Rad1 reduces its association with DNA but does not affect other protein properties. Together, these findings suggest a new regulatory mode for Rad1-Rad10 whereby sumoylation promotes Rad1's DNA dissociation.

Several lines of evidence support the idea that Rad1 sumoylation occurs at DNA lesion sites. First, only a small fraction of Rad1 is sumoylated and only DNA damaging agents that elicit Rad1-mediated repair enhance its sumoylation (Figure 1A). These results hint that the timing of Rad1 sumoylation is strictly regulated. Second, and importantly, sumoylation of Rad1 largely depends on proteins that recruit it to DNA lesion sites (Figure 1B–D). These data strongly suggest that Rad1 needs to be present at sites of damage in order to be sumoylated. Third, Rad1 sumoylation exhibited a strong dependence on Slx4 (Figure 1C), which affects the nucleolysis step after Rad1 recruitment. This further delineates the timing of Rad1 sumoylation to an Slx4-dependent step after lesion recruitment. Finally, nuclease-dead Rad1 that is trapped at lesion sites is hypersumoylated (Figure 1E and F, suggesting that sumoylation does not require catalysis *per se* but rather increases as the inactive protein associates longer with DNA.

Rad1 sumoylation requires either of the two homologous Siz SUMO ligases *in vivo* or *in vitro* (Figure 2). The lack of specific ligase could mean that the SUMO E2 plays important roles in determining the modification site. Mass spectrometry identification of the sumoylation site, as well as *in vitro* and *in vivo* confirmation (Figure 3B and C and Supplementary Figure S2), show that Rad1 is sumoylated at K32, which is within the E2 substrate-recognition site (67). The K32 residue is located outside the Rad1 nuclease and Rad10-binding domains, but is within a domain implicated in DNA association in XPF (68). This location fits with the proposed effect of sumoylation (see below).

The nonsumoylatable *rad1-K32R* mutant exhibited moderate UV sensitivity only at high doses (Figure 3D and E, suggesting that sumoylation of Rad1 may be particularly useful when large numbers of lesions are present. Consistent with this notion, *rad1-K32R* sensitized *tdp1Δ* and *apn2Δ* at high doses of CPT (Figure 3F). This phenotype bias toward large lesion loads is in line with normal repair of a single DNA break in *rad1-K32R* cells (Figure 3G and H. A simple explanation of the phenotype is that high lesion loads demand the efficient recycling of the enzyme, a feature not so important for the repair of a single lesion.

Our biochemical results show that while sumoylation does not alter Rad1 protein levels, protein interactions or nuclease activity, it causes a decrease in DNA binding affinity (Figures 4 and 5), consistent with the location of the modified lysine in a putative DNA association domain. Integrating these biochemical findings with our other results, a plausible model is that sumoylation facilitates the disengagement of Rad1 from DNA after nuclease cleavage (Figure 5F). Although sumoylation of Rad1 reduced binding to both dsDNA (product) and Y-forms (substrate) *in vitro*, only the former likely has functional consequences *in vivo*, as Rad14 and Saw1 may dictate Rad1-Rad10 binding to DNA substrates. We envision that alterations occur in the following steps mediated by Slx4, such that Rad1-Rad10 can better access the substrate DNA and become amenable for sumoylation. Subsequently, sumoylation could impact Rad1-Rad10's affinity for the dsDNA product of the reaction, facilitating its release, thus contributing to the timely repair of large numbers of lesions. Our data do not exclude other possible effects of Rad1 sumoylation in DNA repair. For example, sumoylation could reduce the interaction of Rad1-Rad10 with dsDNA at other regions, thus providing a ‘sweeping’ mechanism to disfavor unproductive DNA association or impedance of other repair pathways, as suggested by the genetic interaction with *apn2Δ*. As Rad1 is not an abundant protein but is required for multiple repair pathways, ensuring high turnover may well be important for Rad1 function in the cell. Further biochemical work is needed to elucidate how sumoylation of Rad1 promotes its dissociation from DNA; for example, sumoylation may induce a conformational change in the N-terminal domain of Rad1 leading to decreased DNA binding ability, similar to the case of sumoylation of TDG (thymine-DNA glycosylase), a human base excision repair enzyme (74,75).

The mild effect of sumoylation on Rad1 function seen here is not an exception, rather it adds to a growing list of cases where sumoylation of DNA repair substrates has moderate but functional effects (53,76–81). These accumulating findings point to the possibility that sumoylation wields a strong influence on DNA repair by collectively exerting small changes on the functions of many proteins. We thus propose that at least part of the SUMO-dependent DNA damage response is mediated by a ‘group effort’ where SUMO moderately alters many protein functions all at once, rather than by a ‘star effect’ hinging on only a few key targets. These two strategies have different biological implications: the former offers a well-buffered and, therefore, robust system for coping with large numbers of DNA lesions, whereas the latter is more sensitive to perturbations and also implies a waste of cellular resources as more substrates are modified than necessary. Importantly, we highlight that the ‘group effort’ strategy entails diverse functional alterations of protein-DNA or protein–protein interactions or enzymatic activities, and not solely by providing molecular glue. Further studies of additional sumoylation substrates will be able to thoroughly test the above notions.

In summary, our results reveal a new contribution of sumoylation to achieve efficient DNA repair by targeting Rad1, and suggest a new way to regulate nucleases by sumoylation. Considering that XPF, the human homolog of Rad1, is sumoylated upon stress (10), and plays multiple important roles in DNA repair, it will be interesting to determine whether sumoylation makes similar functional contributions in human cells.

## SUPPLEMENTARY DATA

Supplementary Data are available at NAR Online.

SUPPLEMENTARY DATA
