# Supplementary material for: Sumoylation of the Rad1 nuclease promotes DNA repair and regulates its DNA association
Source: Nucleic Acids Res. 2014 Apr 20;42(10):6393–404. doi: 10.1093/nar/gku300 (PMC4041466; doi:10.1093/nar/gku300)
Supplement: SUPPLEMENTARY DATA [file supp_42_10_6393__index.html]

Sumoylation of the Rad1 nuclease promotes DNA repair and regulates its DNA association — Sumoylation of the Rad1 nuclease promotes DNA repair and regulates its DNA association — SUPPLEMENTARY DATA 

# Sumoylation of the Rad1 nuclease promotes DNA repair and regulates its DNA association

## SUPPLEMENTARY DATA

**Files in this Data Supplement:**

- SUPPLEMENTARY DATA
