# Supplementary material for: Sumoylation of the Rad1 nuclease promotes DNA repair and regulates its DNA association
Source: Nucleic Acids Res. 2014 Apr 20;42(10):6393–404. doi: 10.1093/nar/gku300 (PMC4041466; doi:10.1093/nar/gku300)
Supplement: SUPPLEMENTARY DATA [file supp_gku300_nar-03640-x-2013-File007.pdf]

Supplementary figures

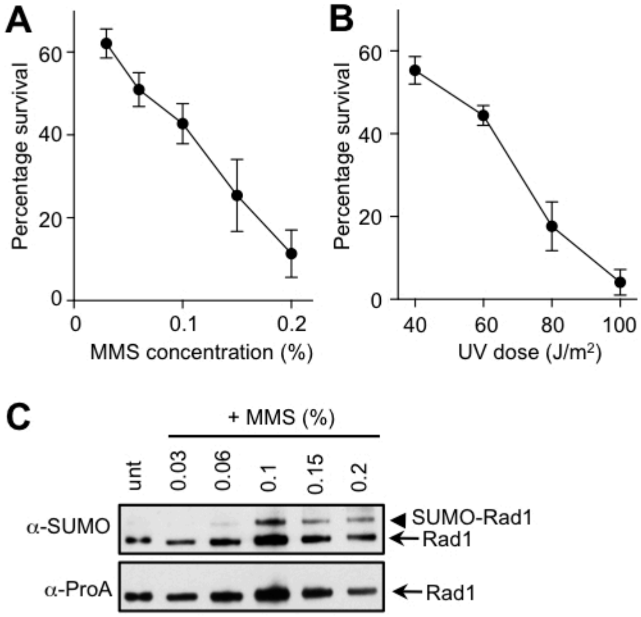

Supplementary Figure 1. Determination of cell survival and Rad1 sumoylation upon genotoxin treatment. (A-B) *Rad1-TAP* cells were subjected to 2 h MMS treatment (A) or UV irradiation (B) and survival percentages were calculated based on three independent trials. **(C)** Rad1 sumoylation is induced after treatment with different MMS doses. *Rad1-TAP* cells were subjected to MMS treatment as in (A) and assessed for sumoylation as in Figure 1A. unt: untreated.

A

1 MSQLFYQGDS DDELQEELTR QTTQASQSSK IKNEDEPDDS NHLNEVENED
51 SKVLDDDAVL YPLIPNEPDD IETSKPNIND IRPVDIQLTL PLPFQQKVVE
101 NSLITEDALI IMGKGLGLLD IVANLLHVLA TPTSINGQLK RALVLVLNAK
151 PIDNVRIKEA LEELSWFSNT GKDDDDTAVE SDDELFERPF NVVTADSLSI
201 EKRRKLYISG GILSITSRIL IVDLLSGIVH PNRVTGMLVL NADSLRHNSN
251 ESFILEIYRS KNTWGFIKAF SEAPETFVME FSPLRTKMKE LRLKNVLLWP
301 RFRVEVSSCL NATNKTSHNK VIEVKVSLTN SMSQIQFGLM ECLKKCIAEL
351 SRKNPELALD WWNMENVLDI NFIRSIDSVM VPNWHRISYE SKQLVKDIRF
401 LRHLLKMLVT SDAVDFFGEI QLSLDANKPS VSRKYSESPW LLVDEAQLVI
451 SYAKKRIFYK NEYTLEENPK WEQLIHILHD ISHERMTNHL QGPTLVACSD
501 NLTCLELAKV LNASNKKRGV RQVLLNKLKW YRKQREETKK LVKEVQSQDT
551 FPENATLNVS STFSKEQVTT KRRRTRGASQ VAAVEKLRNA GTNVDMEVVF
601 EDHKLSEEIK KGSGDDLDDG QEENAANDSK IFEIQEQENE ILIDDGDAEF
651 DNGELEYVGD LPQHITTHFN KDLWAEHCNE YEYVDRQDEI LISTFKSLND
701 NCSLQEMMPS YIIMFEPDIS FIRQIEVYKA IVKDLQPKVY FMYYGESIEE
751 QSHLTAIKRE KDAFTKLIRE NANLSHHFET NEDLSHYKNL AERKLKLSKL
801 RKSNTRNAGG QQGFHNLTQD VVIVDTREFN ASLPGLLYRY GIRVIPCMLT
851 VGDYVITPDI CLERKSISDL IGSLQNNRLA NQCKKMLKYY AYPTLLIEFD
901 EGQSFSLEPF SERRNYKNKD ISTVHPISSK LSQDEIQLKL AKLVLRFPTL
951 KIIWSSSPLQ TVNIILELKL GREQPDPSNA VILGTNKVRS DFNSTAKGLK
1001 DGDNESKFKR LLNVPGVSKI DYFNLRKKIK SFNKLQKLSW NEINELINDE
1051 DLTDRIYYFL RTEKEEQEQE STDENLESPG KTTDDNALHD HHNDVPEAPV

B

Protein	Mass	Score	Coverage	Queries matched
Rad1 (RADiation sensitive 1)	126862	53959	57	1179

Sequence	Modifications	Search Engine Rank	# Missed Cleavages	Observed	Mr (expt)	Mr (calc)	ppm	Score	Expect
K.IKNEDEPDDSNHLNEVENEDSK.V	Sumo	1	1	1018	3053.34	3053.33	0.93	84	4e-09

Supplementary Figure 2. Lysine 32 represents the major SUMO-conjugation site on Rad1. (A) Sequence coverage (red) of Rad1. The two lysines identified as potential sumoylation sites are marked in blue. **(B)** Sequence of top-ranking Rad1 peptide with sumoylated lysine 32 shown in red. Note that the other lysine has a low score.

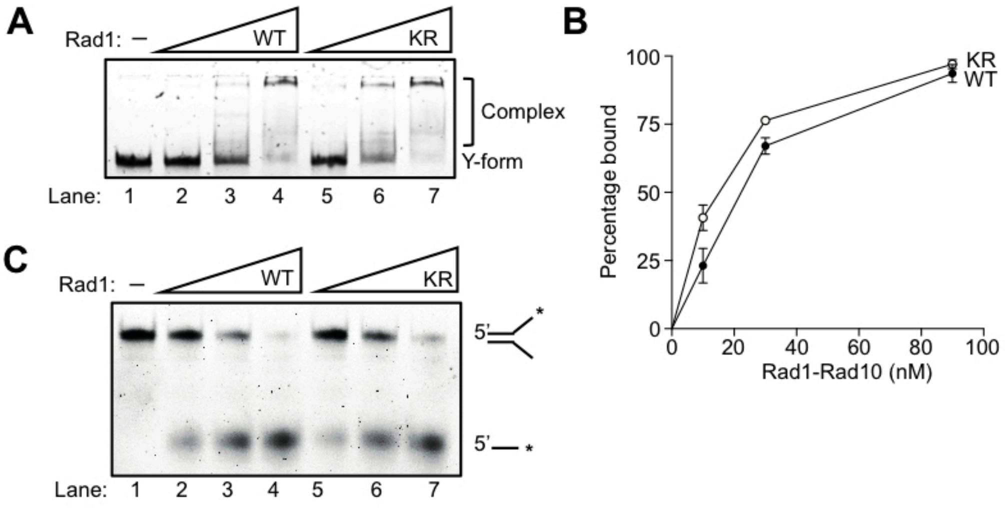

Supplementary Figure 3. Examination of Rad1-K32R for DNA binding and nuclease activity. (A-B) Wild-type Rad1 (lanes 2-4) and Rad1-K32R (lanes 5-7) proteins (10-90 nM) in complex with Rad10 were examined for binding to fluorescently labeled Y-form DNA (4 nM). Products were resolved on a 10% polyacrylamide gel (A), and quantification of several trials is shown in (B). **(C)** Wild-type Rad1 (lanes 2-4) and Rad1-K32R (lanes 5-7) proteins (0.06-1.2 nM) complexed with Rad10 were assayed for nuclease activity on fluorescently-labeled Y-form DNA as in Figure 5B.

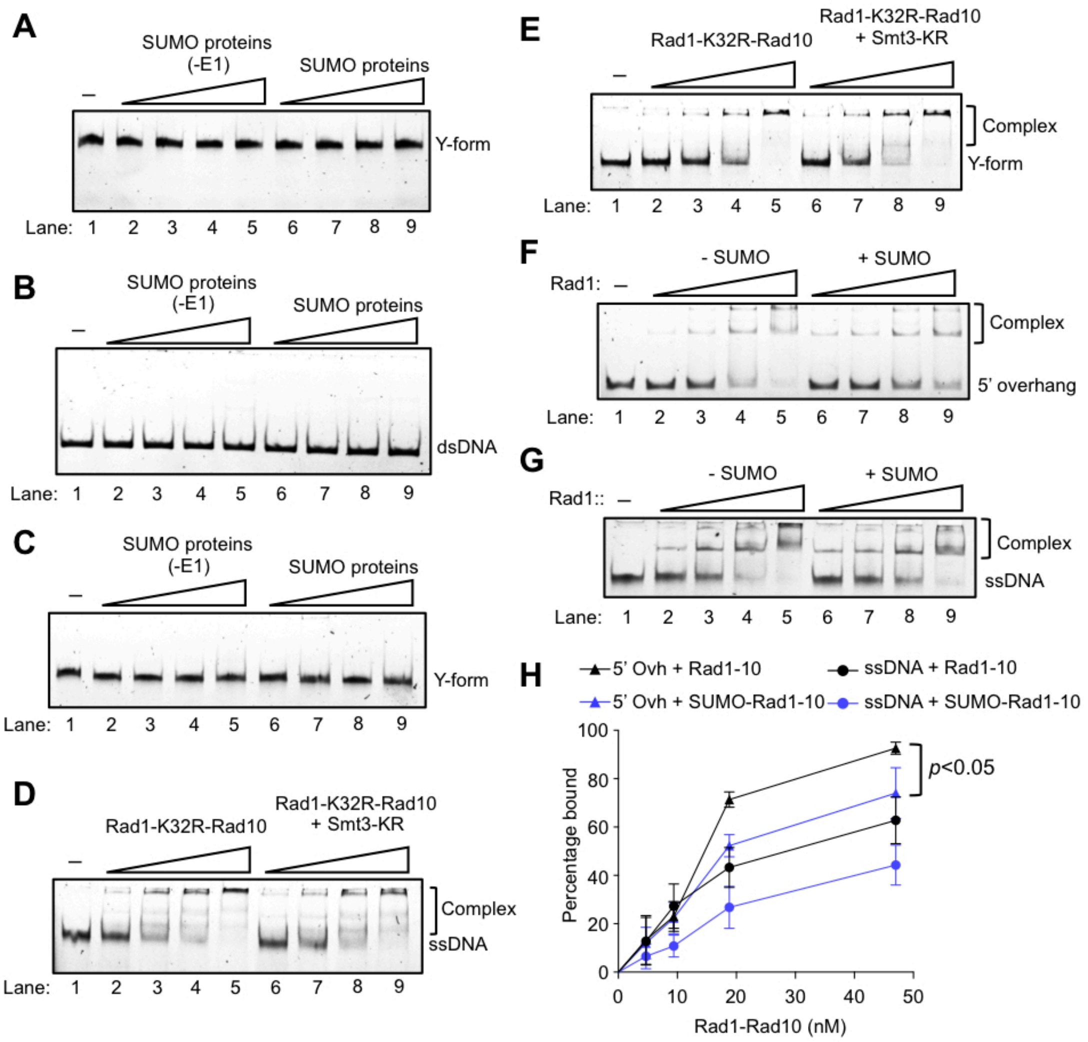

Supplementary Figure 4. Examination of sumoylation mix for DNA binding and nuclease activity and sumoylated Rad1-Rad10 for binding to 5' overhangs and ssDNA. (A-C) Increasing concentrations of SUMO proteins with (lanes 6-9) or without (lanes 2-5) SUMO E1 were analyzed for cleavage of Y-form (A), binding to dsDNA (B) and Y-form (C) as in Figure S3. **(D-E)** Increasing concentrations of Rad1-K32R-Rad10 with (lanes 6-9) or without (lanes 2-5) Smt3-KR (SUMO with lysines mutated to prevent chain formation) were analyzed for binding to ssDNA (D), Y-form DNA (E) and 5' overhang (F) as in Figure S3. **(F-H)** Increasing concentrations of sumoylated (lanes 6–9) and non-sumoylated (lanes 2–5) Rad1 in complex with Rad10 proteins (0.06- 1.2 nM) were tested for binding to 5' overhangs (F) and ssDNA (G). Quantification of several binding trials is shown in (H); 5' Ovh and Rad1-10 denote 5' overhangs and Rad1-Rad10 respectively. The bracket indicates statistically significant difference.
